# Prevention and Control Strategies to Counter Zika Virus, a Special Focus on Intervention Approaches against Vector Mosquitoes—Current Updates

**DOI:** 10.3389/fmicb.2018.00087

**Published:** 2018-02-08

**Authors:** Raj K. Singh, Kuldeep Dhama, Rekha Khandia, Ashok Munjal, Kumaragurubaran Karthik, Ruchi Tiwari, Sandip Chakraborty, Yashpal S. Malik, Rubén Bueno-Marí

**Affiliations:** ^1^ICAR-Indian Veterinary Research Institute, Izatnagar, Bareilly, India; ^2^Division of Pathology, ICAR-Indian Veterinary Research Institute, Izatnagar, Bareilly, India; ^3^Department of Biochemistry and Genetics, Barkatullah University, Bhopal, India; ^4^Central University Laboratory, Tamil Nadu Veterinary and Animal Sciences University, Chennai, India; ^5^Department of Veterinary Microbiology and Immunology, College of Veterinary Sciences, UP Pandit Deen Dayal Upadhayay Pashu Chikitsa Vigyan Vishwavidyalay Evum Go-Anusandhan Sansthan, Mathura, India; ^6^Department of Veterinary Microbiology, College of Veterinary Sciences and Animal Husbandry, Agartala, India; ^7^Division of Biological Standardization, ICAR-Indian Veterinary Research Institute, Izatnagar, Bareilly, India; ^8^Laboratorios Lokímica, Departamento de Investigación y Desarrollo (I+D), Valencia, Spain

**Keywords:** Zika virus, Zika fever, mosquito management, prevention, vector control, arbovirus

## Abstract

Zika virus (ZIKV) is the most recent intruder that acquired the status of global threat creating panic and frightening situation to public owing to its rapid spread, attaining higher virulence and causing complex clinical manifestations including microcephaly in newborns and Guillain Barré Syndrome. Alike other flaviviruses, the principal mode of ZIKV transmission is by mosquitoes. Advances in research have provided reliable diagnostics for detecting ZIKV infection, while several drug/therapeutic targets and vaccine candidates have been identified recently. Despite these progresses, currently there is neither any effective drug nor any vaccine available against ZIKV. Under such circumstances and to tackle the problem at large, control measures of which mosquito population control need to be strengthened following appropriate mechanical, chemical, biological and genetic control measures. Apart from this, several other known modes of ZIKV transmission which have gained importance in recent past such as intrauterine, sexual intercourse, and blood-borne spread need to be checked and kept under control by adopting appropriate precautions and utmost care during sexual intercourse, blood transfusion and organ transplantation. The virus inactivation by pasteurization, detergents, chemicals, and filtration can effectively reduce viral load in plasma-derived medicinal products. Added to this, strengthening of the surveillance and monitoring of ZIKV as well as avoiding travel to Zika infected areas would aid in keeping viral infection under check. Here, we discuss the salient advances in the prevention and control strategies to combat ZIKV with a focus on highlighting various intervention approaches against the vector mosquitoes of this viral pathogen along with presenting an overview regarding human intervention measures to counter other modes of ZIKV transmission and spread. Additionally, owing to the success of vaccines for a number of infections globally, a separate section dealing with advances in ZIKV vaccines and transmission blocking vaccines has also been included.

## Introduction

Immediately after the Ebola virus threats, Zika virus (ZIKV) has acclaimed to be the most recent virus that is threatening the global human population. After remaining innocuous for nearly six decades (since its first report in the year 1947), its emerging status propelled to declare it as an emergency situation (Public Health Emergency of International Concern) on February 1, 2016 by the World Health Organization (WHO) (Chang et al., 2016; Chen and Hamer, 2016; Gulland, 2016; Singh et al., 2016, 2017). ZIKV, a mosquito-borne virus, belongs to the Spondweni serocomplex under the genus *Flavivirus*, family *Flaviviridae*. ZIKV causes a dengue-like febrile illness which is mild in nature, but its recent association with neurological complications (microcephaly in newborns and Guillain Barré Syndrome) has worsened the circumstances (Chitti et al., 2016; Armstrong et al., 2017). Initially the virus was restricted to Africa and Asia, but later acquired status of global threat spreading worldwide (Chang et al., 2016; Chitti et al., 2016; Singh et al., 2016; Vest, 2017; Zhang et al., 2017). The emergence of ZIKV, owing to its rapid spread, attaining higher virulence and affecting large human population with complex clinical manifestations across different countries, created panic and frightening situation to public. Additionally, lack of advanced knowledge on ZIKV and dearth of suitable prevention and treatment strategies aggravated the situation across the globe. Alike other flaviviruses, the principal mode of ZIKV transmission is by mosquitoes. Hitherto reports have confirmed that both sylvatic (between vector: hematophagous mosquito and primary host: vertebrates) and urban transmission cycles plays a crucial role in the spread of ZIKV, resulting in epidemics many a times (Diallo et al., 2014; Chan et al., 2015, 2016).

Owing to the high public health concerns associated with this virus, researchers across the globe are working high in studying in-depth the virus and the disease it causes. Appreciable focus has been made on designing and developing effective diagnostics, drugs, prophylactics, vaccines and devising appropriate prevention and control strategies (Singh et al., 2016; Munjal et al., 2017a; Rather et al., 2017). Using state-of-art technologies, quick and reliable diagnostics have been developed for detecting ZIKV infection (Singh et al., 2018), while several drug/therapeutic targets along with vaccine candidates have become known (Fernandez and Diamond, 2017; Munjal et al., 2017a,b; Sharma and Lal, 2017). Recently, viral NS2B-NS3 protease was found to be an effective antiviral drug target for the treatment of ZIKV (Kang et al., 2017). Despite these advances, currently there is neither any effective treatment nor any vaccine available against ZIKV and its associated complications. Under such circumstances, adapting suitable prevention and control measures such as checking the spread and bite of the vector mosquitoes through controlling their population by means of mechanical, chemical, biological and genetic approaches, remains crucial and valuable steps to keep ZIKV infection under check (von Seidlein et al., 2017). Apart from this, several other known modes of ZIKV transmission which have gained significance in the recent past such as intrauterine, sexual intercourse, and blood-borne spread need to be checked and kept under control through following appropriate precautions and utmost care during sexual intercourse, blood transfusion and organ transplantation. The virus inactivation by pasteurization, detergents, chemicals and filtration can effectively reduce viral load in plasma-derived medicinal products. Added to this, strengthening of the surveillance and monitoring of ZIKV, enhancing biosecurity as well as avoiding travel to Zika infected areas would aid in keeping ZIKV infection under check. Apart from these effectual measures, predisposing factors including climate changes (global warming), modulation of population dynamics, rapid globalization, fast travel aids and pathogen related attributes, which are responsible for an upsurge in the incidences, outbreaks and emergence of mosquito-borne diseases especially caused by pathogens like Chikungunya, Dengue, Japanese encephalitis, West Nile, and Zika virus need adequate attention (Dhiman et al., 2010; Dhama et al., 2013; Parham et al., 2015; Gautret and Simon, 2016).

Here, we describe the salient advances in prevention and control strategies to combat ZIKV with a focus on highlighting various intervention approaches against the vector mosquitoes of this viral pathogen as well as an overview on human intervention measures needed to counter other modes of ZIKV transmission and spread. The vaccine platforms that are being intended for the development of an effective vaccine against ZIKV and other transmission blocking vaccines are also dealt herein.

### Modes of transmission of ZIKV

**Figure 1:**
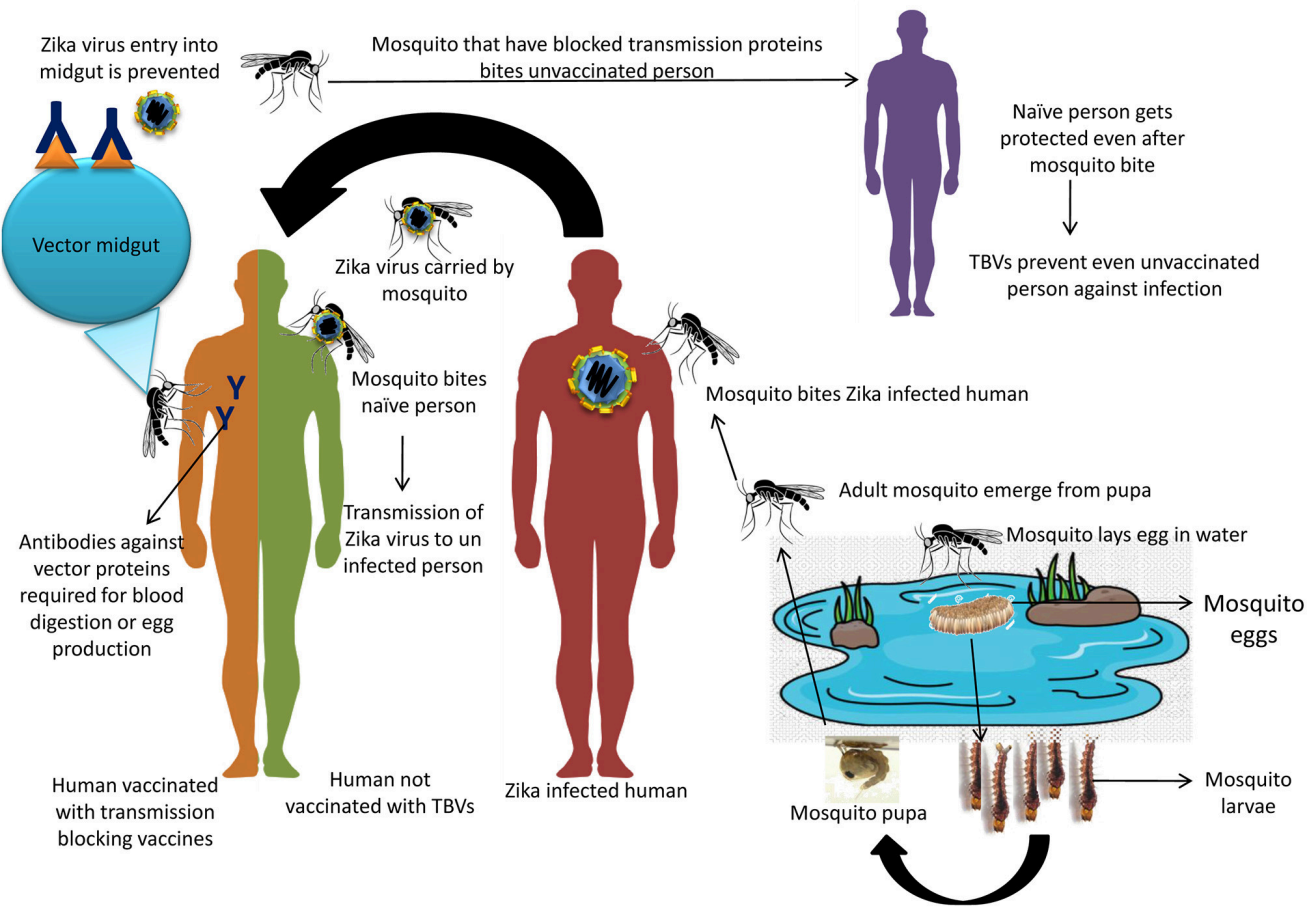
Modes of ZIKV transmission between infected and uninfected person through mosquito bite and possible mode of TBVs in prevention and control of ZIKV infection.

The well-established mode of ZIKV transmission is through mosquito bite. The most significant mosquito vector responsible for transmission of ZIKV is *Aedes* (*Stegomyia*), but *Anopheles, Culex, Mansonia*, and *Eretmapodites* can also transmit the virus (Berthet et al., 2014; Diagne et al., 2015). ZIKV was first isolated from *Aedes africanus*, which plays a crucial role in the ZIKV sylvatic transmission cycle. *Aedes aegypti* and *Aedes albopictus* are more significant vectors in the urban transmission cycle, as they are distributed over a wider geographical range (Gasperi et al., 2012; Chan et al., 2016). Figure 1 depicts the cycle of ZIKV transmission between infected and uninfected humans through the mosquito bites. The reservoir host-mosquito-reservoir host transmission cycle has a period of incubation that varies from 2 to 5 days in the reservoir host and 5 to 7 days in the mosquito (Al-Qahtani et al., 2016). Non-vectored transmission can occur through blood transfusion, transplacental, and sexual modes (Mead et al., 2017). Other means of virus transmission include breast feeding, saliva, or urine, however no concrete evidence exists regarding ZIKV transmission through these routes (Foy et al., 2011; Musso et al., 2014). Additional in-depth studies are warranted to find the probability of alternate mode of ZIKV transmission. Presently, these non-vector borne routes have not gained as much research attention as the mosquito-borne route. The strategies that have been adopted for *Ae. aegypti* eradication remain inefficient as is indicated by a very high mosquito infestation index (Zanluca and dos Santos, 2016). It appears that these strategies have been clearly formulated as a public health response to a ZIKV epidemic. These strategies involve intercepting the life cycle (enzootic) to halt the growth of the vector in its native environment, reducing exposure of susceptible subjects to the vector, limiting the source of the vector to the urban population, eliminating the vector, and preventing infected mosquitoes from biting humans (Weaver, 2013).

## Prevention and control strategies countering the Zika virus

Prevention is always considered better than cure. Strategies to prevent ZIKV infection include mechanical, chemical, or biological control measures to prevent mosquitoes from spreading. Correspondingly, personal preventive measures are parallelly important.

### Prevention and control of mosquitoes

Since, the prime mode of ZIKV transmission is through mosquitoes, it is essential to concentrate on the methods that can control mosquitoes, so that its spread can be kept at bay. Lately, several preventive and control measures are reported to check the ZIKV spread (von Seidlein et al., 2017). These include mosquito population control through preventing or inhibiting breeding (Hajra et al., 2016), and protecting the community from the infected *Aedes* vector population. For effective mosquito control, mechanical, chemical, and biological measures should be employed (Araújo et al., 2015; Malone et al., 2016). In this direction, the herbal remedies and biological control strategies, such as the release of sterile male *Aedes* have been employed (Singh et al., 2016). It is crucial that public health authorities must intervene now because the virus has spread over larger areas rather than concentrating in a single geographic location (Aubry et al., 2015).

#### Mechanical control measures

Mechanical control measures are the age old techniques, which have been adopted and practiced out in several countries; since these are easy and cost-effective methods for the control of mosquito population. Mechanical control measures include the removal of all objects storing unwanted water, as these become sites for mosquitoes breeding. Streets should be properly cleaned and buildings and housing units must be properly maintained. Personal and community hygiene is also a major part of this technique, which can reduce mosquito breeding places. The use of mosquito nets on windows and mosquito-proof water storage options should be encouraged (Sikka et al., 2016). Ovitraps are cheaper and maintenance free, which can be used to reduce mosquito population (Barrera et al., 2014). Major urban ZIKA vectors, namely *Ae. aegypti* and *Ae. albopictus* show a strong degree of domiciliary behavior and are found breeding in different types of artificial containers placed in private areas. Therefore, citizen awareness is essential to avoid potential breeding sites in domiciles and this mechanical control measure can be easily applied even by a non-qualified personal. Following a better hygienic life style, mosquito bites / breeding sites can be eliminated and thereby major threats of vector borne diseases like ZIKV can be prevented.

#### Chemical control measures

Chemical treatment includes the use of pyrethroids, organochloride, and organophosphorus, which primarily act on the nervous system of the vector (van den Berg et al., 2012). Imidacloprid, thiacloprid, and thiamethoxam have larvicidal and adulticidal efficacies in different mosquito species. The use of fogging with insecticides is adopted in the outdoor environment to control the vector population though this can lead to development of resistance among the vector population (Maciel-de-Freitas et al., 2014; von Seidlein et al., 2017). The development of resistance and bioaccumulation are major problems associated with the use of chemicals to control mosquitoes (Uragayala et al., 2015). Moreover, most of these chemical products have other environmental problems because of their wide range of toxic action for several organisms, such as being non-specific insecticides and negatively affecting in some cases to all arthropods or even the rest of aquatic fauna as well as mammals and birds. Consequently, although these chemicals are currently necessary to reduce adult mosquito populations in concrete epidemiological scenarios, however a deep analysis of benefits-costs balance should be addressed before implementing a large-scale use of these insecticides for mosquito control. Repellents can be used at household to prevent mosquito nuissance at home thus preventing major mosquito borne diseases like ZIKV. Recently, a study compared the efficacy of repellet sprays with devices that repell mosquitoes, where N,N-Diethyl-meta-toluamide (DEET) and p-menthane-3,8-diol were found better effective repellent among the 11 different mosquito repellent sprays used (Rodriguez et al., 2017). Similarly, devices with metofluthrin were found more effective in attracting mosquito among the five devices compared (Rodriguez et al., 2017). Of note, DEET and picaridin-containing insect repellents have been found safe for the pregnant women (Kline and Schutze, 2016). Permethrin treated mosquito repellent apparels are available to prevent from mosquito bites (Richards et al., 2017). To note further, unwashed clothes and those which were not exposed to light showed highest repelling activity (Richards et al., 2017).

Insect growth regulators (IGRs) are promising alternatives with high degrees of insecticidal efficacy and environmentally safe compared to conventional chemical larvicides (Mulla et al., 1986; World Health Organization, 1997). Analogs of juvenile hormone like methoprene or pyriproxyfen, and chitin synthesis inhibitors such as diflubenzuron and triflumuron are some examples of efficient mosquito larvicides. Some of these IGRs like pyriproxyfen are good candidates for autodisemination strategies, having positive results in recent trials conducted against ZIKV vectors (Unlu et al., 2017; von Seidlein et al., 2017). Since essential endocrine pathways for the activity of pyriproxyfen are lacking in human, there is no negative effect in human population (Hirano et al., 1998). Notingly, the judictive usage of appropriate chemicals could prevent the growing mosquito population and spread of several diseases inclduing ZIKV infection.

#### Biological control measures

Alternative to the use of chemicals, several biological measures against mosquitoes have been exploited with few having high efficacy to combat mosquito population at large. Numerous biological measures, such as the use of bacteria, fungi, plants, and fish employed to control the growth and propagation of the mosquito population.

##### Biocontrol measures using bacteria

A bacterium that can infect mosquitoes could be exploited to manipulate mosquito population. In 1976, the bacteria *Bacillus thuringiensis* subsp. *israelensis* (Bti) was isolated and found to be toxic to mosquito larvae (Goldberg and Margalit, 1977), and since the early 1980s, Bti-based insecticides have been available commercially. Bti is a target-specific insecticide that at the time of sporulation produces a highly specific delta-endotoxin, which is only toxic to larvae of mosquitoes, black flies and closely related flies upon ingestion. *Bacillus sphaericus* (Bs) is another bacterium commonly used against mosquitoes with similar characteristics to Bti. However, Bs that has been shown to persist longer than Bti in polluted habitats and, under certain circumstances, can recycle in larval cadavers increasing the residuality (Lacey, 2007). Both types of bacterial biolarvicides have become the predominant non-chemical means employed in recent years for control of mosquito larvae at several regions of USA and many countries of Europe.

Another strategy employed is the use of intracellular bacteria *Wolbachia*, which has been used as a biopesticide to control mosquito population. The method of use of *Wolbachia* for suppression of mosquito population is called as Incompatible Insect Technique (Lees et al., 2015). The intracellular, maternal transferring bacterium *Wolbachia* can effectively reduce vector competence for ZIKV by reducing the lifespan of the female mosquito and inducing cytoplasmic incompatibility (Nguyen et al., 2015; Aliota et al., 2016). Though *Wolbachia* is found in several nematodes and arthropods they do not naturally occur in *Ae. aegypti* (Werren, 1997; Walker et al., 2011). When *Wolbachia* infected female mosquito mates with normal or *Wolbachia* infected male, offspring's with *Wolbachia* are produced. Similarly, when normal female mates with *Wolbachia* infected male, offspring's will not be produced owing to cytoplasmic incompatibility (Caragata et al., 2016). *Wolbachia*-infected mosquitoes were released in areas of Rio de Janeiro during recent ZIKV episodes to control mosquitoes and virus transmission (Callaway, 2016). *Ae. aegypti* infected with the *Wolbachia*, a Drosophila strain, efficiently blocks the transmission of ZIKV. The male mosquitoes once infected with *Wolbachia* prior to mating results in laying of sterile eggs (Dutra et al., 2015). Alpha-proteobacteria of the genus *Asaia* are acetic acid symbionts present in the female gut and the male reproductive tract of adult *Ae. aegypti* and anopheline mosquitoes. The bacteria are both horizontally and vertically transmitted and can be engineered to reduce the lifespan of the insect. *Wolbachia* sp. and *Asaia* sp. may negatively interact and are mutually exclusive (Favia et al., 2008; Lambrechts et al., 2015; Rossi et al., 2015). Bacteria are easy to grow, maintain and manipulate in order to harbor desirable qualities; and on appropriate culture media, bulk amounts may be generated with relative ease. Thus, utilization of the predator-prey approach can help to avert the transmission of ZIKV.

##### Biocontrol measures using fungi

The fungi, *Metarhizium anisopliae* and *Beauveria bassiana*, can also be used as biocontrol measures against mosquitoes. Their conidia can adhere to and germinate on the cuticle and penetrate into the body of *Aedes* mosquitoes. The hyphae thicken and disrupt the integument and hemocoel. This is followed by invasion of other internal organs and death of the host, which leads to further dispersal of conidia to infect other insects (Darbro and Thomas, 2009; Tiago et al., 2014). The fungus, *Beauveria bassiana*, has been approved by the United States Environmental Protection Agency to be used as a biological mosquito control measure. Jaber et al. (2016) isolated 42 fungal strains from 17 different decaying arthropod cadavers. Out of theses 42 isolates, 8 isolates exhibited high pathogenicity in a *Drosophila melanogaster* model. Only one strain, *Aspergillus nomius*, exhibited properties similar to *Beauveria bassiana* and killed 100% of the *Aedes* adults. Hence, it was suggested that *A. nomius* can also be used for the control of mosquitoes. *B. bassiana* had already shown better adulticidal activity and hence can be used in the mosquito traps for an effective control of the vectors (Snetselaar et al., 2014). Insect cuticle is the major barrier that primarily prevents the entry of pathogens; but the cuticle is usually breached by the germinating fungal spores, and in real terms, the fungus seems to be the true entomopathogen, however, to reach to any conclusion, it is required to be tested in the natural infection settings. This may be obtained by simply spraying the fungal spores and then evaluating the efficacy of fungus to be used as a biocontrol measure.

##### Biocontrol measures using mosquitoes against mosquitoes

**Figure 2:**
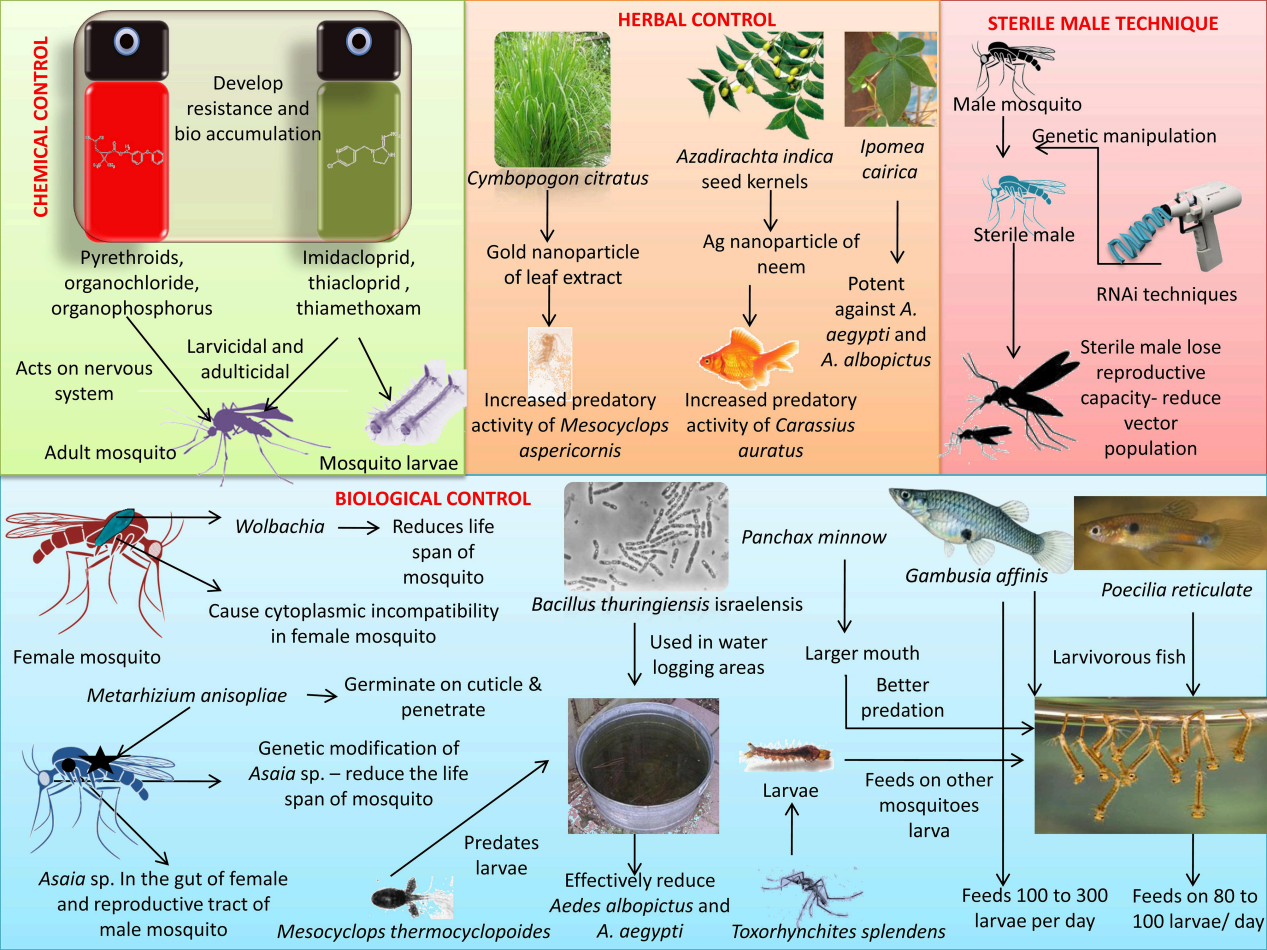
Different mosquito vector control strategies for preventing transmission of Zika virus. Control methods include chemical (use of repellents), biological (use of bacteria, fish, etc.), herbal and sterile male techniques.

Certain mosquitoes predates other mosquito larvae and even adults, hence these species can be utilized to control *Aedes* sp. mosquitoes that spread ZIKV. *Toxorhynchites splendens* is a species of mosquito that does not feed on blood. Its larvae feed on the larvae of other mosquito species, while the adults feed on honeydew, fruit, and nectar (Benelli et al., 2016a). In a small-scale study, *Mesocyclops aspericornis* and *Toxorhynchites speciosus* together were found to contain the *Aedes* sp. population and together they formed a compatible predator pair, with one not affecting the survival of the other (Brown et al., 1996). *Toxorhynchites* adults are often called elephant mosquitoes because they are larger than *Aedes* mosquitoes. They are considered harmless to humans due to their non-hematophagous nature. *Berberis tinctoria* fabricated silver nanoparticles exhibit acute toxicity toward *Ae. albopictus*, while sparing the mosquito predators *T. splendens* and *M. thermocyclopoides* (Kumar et al., 2016). Hence, such green nanoparticles may play an effective role as eco-friendly nanopesticides. However, the effects on other aquatic fauna need to be evaluated. Zuharah et al. (2015) showed a prey preference of *T. speciosus* toward *Ae. aegypti*, when *Ae. aegypti, Ae. albopictus*, and *An. sinensis* were present in the same container (Zuharah et al., 2015). Mosquito predators constitute beneficial strategy *in-situ* and after introduction in the water body, these predators provide a sustainable system for mosquito clearance and hence mosquito predators can be used for control of *Aedes* sp. mosquitoes thereby preventing the spread of ZIKV. Various mosquito vector control strategies have been represented in Figure 2.

##### Biocontrol measure using copepods

*Mesocyclops* and *Macrocyclops* are the major Copepods used as mosquito biocontrol measure. These feed on *first* instar larvae. *Mesocyclops thermocyclopoides* is a predator of the *Ae. aegypti* mosquito and its predatory efficiency increases by 8.7% in the presence of *Solanum xanthocarpum* fruit extract (Mahesh Kumar et al., 2012). Simple protocols have been developed for breeding Copepod species for maintenance and mass propagation prior to their release as biocontrol measures (Suarez et al., 1992).

Electron microscopy of predation behavior of *Mesocyclops* sp. demonstrated that the attack occurs first on the anal segment, followed by the siphon and the abdomen. The head segment is the least preferred site of attack (Schaper and Hernández-Chavarría, 2006). Treatment with *Mesocyclops*, reduced the incidence of dengue virus (DENV) outbreak in Vietnam in a program during 2002 and 2003 (Vu et al., 2005). These results sustained for several years, with an abundance of *Mesocyclops* and reduced numbers of *Ae. aegypti* larvae (Kay et al., 2010). Predation efficacy of *M. formosanus* is higher for larvae at the first and second instars than the third and fourth instars. Several other predatory copepods include *Cyclops vernalis, Mesocyclops aspericornis, Mesocyclops edax, Mesocyclops guangxiensis, Mesocyclops longisetus* (Mahesh Kumar et al., 2012; Anbu et al., 2016; Kumar et al., 2016). Practically, rearing a copepod is economic and easy as it requires little assistance for maintaining the colonies. Hence, copepods like *Mesocyclops* can be used for the control of mosquitoes spreading ZIKV.

##### Biocontrol measures using plants

Many plant-derived products are being tested for their effectiveness against mosquitoes. For lavicidal purpose there has been employment of several plants to synthesize nanomosquitocides. A methanolic seed extract from the Brazilian plant, *Myracrodruon urundeuva* Fr. Allemao, was found to be highly toxic to *Ae. aegypti* larvae at the concentration of 1,000 μg/ml. As, the plant extract also showed toxic effects to non-mosquito predator, *Artemia salina* (LC_50_ of 1,500 ppm), due care should be provided when considering this plant extract in the mosquito control program (Souza et al., 2011). Reegan et al. (2015) tested extracts from five medicinal plants, *Aegle marmelos* (Linn.), *Limonia acidissima* (Linn.), *Sphaeranthus indicus* (Linn.), *Sphaeranthus amaranthoides* (Burm. f), and *Chromolaena odorata* (Linn.), for ovicidal activity against *Culex quinquefasciatus* and *Ae. Aegypti*. A hexane extract of *L. acidissima* gave the highest ovicidal activity (79.2 and 60% against *Cx. quinquefasciatus* and *Ae. Aegypti*, respectively) at the concentration of 500 ppm and was found to be effective for use in an integrated mosquito management program (Reegan et al., 2015). Testing of the botanical insecticides extracted from other plants, including *Apium graveolens* (the seed oil is a good repellent against *Aedes* mosquitoes), *Callistemon rigidus*, and *Persea americana* revealed *A. graveolens* to have larvicidal activity against *Ae. aegypti* (LC_50_ and LC_90_ of 16.10 and 29.08 ppm, respectively). Hitherto reports confirm that the oil extract from this plant possess 100% repellent effect against adult mosquiotes upto 3 h (Kumar et al., 2014; Ramkumar and Karthi, 2015).

An essential oil from *Syzygium lanceolatum* showed larvicidal effects on *Ae. aegypti* (LC_50_ value of 55.11 ppm), *Ae. albopictus* (LC_50_ value of 66.71 ppm), *An. stephensi* (LC_50_ value of 51.20 μg/ml), *An. subpictus* (LC_50_ value of 61.34 ppm), *Cx. quinquefasciatus* (LC_50_ value of 60.01 ppm), and *Cx. tritaeniorhynchus* (LC_50_ value of 72.24 ppm) larvae. Its toxic effect on water bugs (*Anisops bouvieri* and *Diplonychus indicus*) and fishes (*Gambusia affinis* and *Poecilia reticulata*) was found to be very low (LC50 value between 4,148 and 15,762 ppm) and hence, this can be used as an eco-friendly mosquito repellent (Benelli et al., 2016b). Essential oils from the *Casuarina equisetifolia* plant have been shown to have mosquitocidal effects against *An. gambiae* and *Ae. aegypti*. Nineteen compounds were identified from the oil, with fatty acids being the major component. Other compounds that were identified include n-hexadecanoic acid (18.67%), cis-13-octadecanoic acid (17.83%), tridecane (11.84%), Undecane (10.45%), Hentriacontane (8.91%), Nonanal (8.62%), and Oxirane (2.43%). The knock down (KDT_50_) potential of the essential oil was evaluated and it was found to be 40 min for *An. Gambiae* while 61 min for *Ae. aegypti*. The essential oil could also effectively kill 100% of the mosquitoes within 24 h (Adeosun et al., 2016). Another study also reported that essential oil from *Origanum scabrum* had effective larvicidal properties against a variety of mosquitoes, including *An. stephensi* (LC_50_ of 61.65 ppm), *Ae. aegypti* (LC_50_ of 67.13 ppm), *Cx. quinquefasciatus* (LC_50_ of 72.45 ppm), and *Cx. tritaeniorhynchus* (LC_50_ of 78.87 ppm) (Govindarajan et al., 2016a). Similarly, the essential oil also had ovicidal activity against *An. stephensi, Ae. aegypti, Cx. quinquefasciatus* and *Cx. tritaeniorhynchus* with LC_50_ value of 160, 200, 240, and 280 ppm, respectively. The essential oil also prevented the bite of *An. stephensi, Ae. aegypti, Cx. quinquefasciatus* and *Cx. tritaeniorhynchus* up to 210, 180, 150, and 120 min, respectively. This essential oil was found less toxic to non-mosquito predators like *Anisops bouvieri, Gambusia affinis*, and *Diplonychus indicus* with LC_50_ range of 4,162 to 12,425 ppm (Govindarajan et al., 2016a). Essential oils from other herbs, such as *Heracleum sprengelianum*, have also been studied for their larvicidal activity, revealing effectiveness against *Ae. albopictus, An. subpictus, Cx. tritaeniorhynchus* with LC_50_ value of 37.5, 33.4, and 40.9 ppm, respectively. This essential oil was found less toxic to fish *A. bouvieri, D. indicus*, and *G. affinis* (Govindarajan and Benelli, 2016). Different plant parts of *Ipomoea cairica* (Railway Creeper) were evaluated for larvicidal efficacy against *Aedes* using the World Health Organization (WHO) standard larval susceptibility test method. An acetone extract of the *I. cairica* leaf was found most potent against *Ae. aegypti* and *Ae. albopictus* with LC_50_ value of 450 ppm (AhbiRami et al., 2014). In the tests conducted according to the WHO test procedures for larval and adult bioassays, *Glycosmis pentaphylla* leaf extracts exhibited larvicidal and adulticidal activities against *An*. *stephensi, Cx*. *quinquefasciatus*, and *Ae*. *aegypti* mosquitoes. The extracts had larvicidal activity with 0.4, 266.9, 58.5 ppm corresponding to *An. stephensi, Cx. quinquefasciatus*, and *Ae. Aegypti* respectively (Ramkumar et al., 2016).

Hexanic extracts from flower of *Clusia fluminensis* contain clusianone, a benzophenone that can significantly inhibit survival and thus kills the larvae of *Ae. aegypti* at a concentration of 50 mg/L (Anholeti et al., 2015). Also, in the presence of plant-synthesized metal or carbon nanoparticles, the predatory efficiency of biological agents increases. Low quantities of gold nanoparticles, synthesized using an extract from the *Cymbopogon citratus* leaf, increased the predatory efficiency of the cyclopoid crustacean, *Mesocyclops aspericornis*, against *Ae. aegypti* from 56 to 77.3% (Murugan et al., 2015a). Similarly, a methanolic extract from the seaweed, *Gracilaria firma*, increased the predation efficacy of *M. formosanus* for *Ae. aegypti* (Kalimuthu et al., 2014). Hence, *Mesocyclops* sp. combined with proven herbal extracts can synergistically increase the predation of *Aedes* sp. Neem cake, a byproduct of neem oil extraction from the seed kernels of *Azadirachta indica*, has also been used to biosynthesize silver nanoparticles (AgNP). Such AgNP showed lesser LC_50_ value of 3.969 ppm (for larva of *Ae. aegypti*) and 8.308 ppm (for pupa) as compared to neem cake having LC_50_ value of 106.53 (larva) and 235.36 ppm (pupa). Treatment with these nanoparticles also increased the predation efficiency of *Carassius auratus* (Goldfish) (Chandramohan et al., 2016). Silver nanoparticle-based *Ichnocarpus frutescens* extract showed larvicidal properties against *An. subpictus, Ae. albopictus*, and *Cx. tritaeniorhynchus*, and hence, is a promising candidate for mosquito control (Govindarajan et al., 2016b). Overall, the use of herbal extracts seems to be a promising area for the elimination of mosquitoes without any problem of bio-accumulation. However, prior to their use, these extracts must be tested for any acute or chronic toxic activity against other insects or higher organisms.

##### Biocontrol measures using fish

The fish, *Gambusia affinis*, is one of the most successful biological control measures for mosquitoes, having been historically introduced in many regions of the world in the fight against malaria with notable results in terms of vector reduction. The fish has a predation rate of 100–300 larvae per day. Immediately after its use for predation of mosquitoes, this fish was found to be invasive as it not only predate mosquito larvae but also predate the native fishes and tadpoles (Mischke et al., 2016). A study conducted on 36 *G. affinis* fish revealed that almost 24 fish had tadpole of *Hyla regilla* (Pacific tree frog) in their stomach while only 20 fish had mosquito larvae. Thus, *G. affinis* does not specifically predate mosquito larvae; hence care should be taken while using this fish specifically for mosquito larvae predation (Goodsell and Kats, 1999). Similarly, another predatory fish *Gambusia holbrooki* was used for mosquito larvae control in Australia but later this fish became a nuisance as it caused the decline of native aquatic life (Wilson, 1960; Walton et al., 2012). A predatory efficacy study was conducted employing three native fish species of Australia (*Pseudomugil signifer, Hypseleotris galii*, and *Pseudogobius* sp.) and *Gambusia holbrooki*. Best results were obtained for *P. signifier* and *G. holbrooki* in predating the mosquito instars, but due to the concern raised over *G. holbrooki*, the native species *P. signifier* was recommended for the mosquito control in Australia (Griffin, 2014). Another fish used for this purpose is the guppy (*Poecilia reticulata*), which consumes about 80–100 mosquito larvae per day. Predatory fishes are small, highly tolerant and can survive in shallow water and water body margins where mosquito larvae breed. Through adequate education on their effectiveness, these fish may be incorporated as part of an integrated mosquito management program (Kant et al., 2013; Sarwar, 2015). *Poecilia reticulata* is able to tolerate higher temperatures and marshy habitats. However, *Panchax minnow* was found to have superior predation efficiency in deep water compared to the guppy. Although the guppy is a more efficient predator in shallow water, overall predation efficiency of *Panchax minnow* is greater, because its mouth is 70% larger than the mouth of a similarly sized guppy (Gupta and Banerjee, 2013). *Gambusia holbrooki* and *P. signifier* fish exhibit high predation rates of both 2nd and 4th instar larvae of *Ae. vigilax* (Griffin, 2014).

*Channa gachua*, an endogeneous species from Assam, India, has been shown to be a voracious carnivorous feeder. It can live in turbid water and therefore, it has a wider biocontrol application. *P. sophore* and *T. fasciata* are omnivorous fish which, in the absence of mosquito larvae, may feed upon algae and plankton (Phukon and Biswas, 2013). *C. gariepinus* is also an exotic fish having higher predation efficiency than *Gambusia affinis, Poecilia reticulata*, and *Carassius auratus* and it is well adapted for controlling mosquitoes in shallow water bodies and marshy areas. *C. gariepinus* has been shown to successfully control a mixed population of *An. stephensi, An. subpictus* Grassi, *Armegeres subalbatus* Coquillett, *Cx. quinquefasciatus*, and *Cx. vishnui* (Ghosh et al., 2005). A recent study has revealed that the combination of larvivorus fish with larvicidal chemicals gave better results when compared with the use of predatory fish alone (Anogwih et al., 2015); however, threat of disturbance of native flora and fauna remain in doubt. Hence, prior identification of native larvivorus fish and combing with larvicidal agents can prevent mosquitoes at their instar stage. Thus, there is need to cautiously select a predatory fish of native species that would not cause damage to the already existing flora and fauna.

##### Biocontrol measures using tadpoles

Tadpoles of frogs are found to predate larvae of different mosquito species. Tadpoles of five frog species namely *Bufo, Euphlyctis, Hoplobatrachus, Polypedates*, and *Ramanella* were studied for the predatory activity of *Ae. aegypti* eggs. Notably, *Ae. aegypti* eggs were found in the guts of all the tadpoles showing predatory activity for mosquito eggs (Bowatte et al., 2013). Consequently, a study revealed that larvivorous property of *Hoplobatrachus tigerinus* tadpole against *Ae. aegypti* increases with the use of synthesized silver nanoparticles and *Artemisia vulgaris* leaves (Murugan et al., 2015b). It was opined that most of the tadpoles are herbivores, which is a contraindicatory finding to the earlier fact. Further, it has been confirmed that both tadpole and mosquito larvae feed on the debris in the aquatic environment (Weterings, 2015) and even certain mosquitoes analyze the aquatic environment for the presence of tadpoles before oviposition, thus adapting to the aquatic environment for its survival (Weterings, 2015). More studies are warranted in this direction so that tadpoles which share the micro-environment with the mosquito larvae can be exploited to control mosquito population.

#### Genetic tailoring

Mosquitoes are genetically tailored to transmit lethal gene to offspring that is kept under the genetic control of tetracycline gene. Hence, offspring's of the genetically tailored mosquitoes need tetracycline in water for their survival; which usually does not exist in environmental water, thus leading to death of the larvae (Specter, 2012; von Seidlein et al., 2017). Field studies in Brazil and Cayman Islands showed 85 and 80% reduction in vector population, respectively (Harris et al., 2012; Achee et al., 2015). Prior approval is also needed from suitable governing bodies before releasing these genetically modified mosquitoes into the environment (Vythilingam et al., 2016). Mosquitoes can be genetically modified, thereby resulting in the development of a mosquito population whose offspring are incapable of surviving. A reduction in the mosquito population through genetic modification will also cause a reduction in mosquito bites in humans and susceptible primates. The genetically engineered strain, *Ae. aegypti* OX513A, has previously been used for controlling the spread of DENV and it is expected to also be effective in controlling the spread of ZIKV (Phuc et al., 2007; Alphey and Alphey, 2014). In fact, genetically engineered *Ae. aegypti* mosquitoes developed by the British firm Oxitec have been approved by the FDA to combat ZIKV, with the expectation that these mosquitoes will have no significant environmental. These genetically engineered mosquitoes will be able to self-limit the mosquito population. Though the genetically modified strain, *Ae. aegypti* OX513A, is intended to curb mosquito vector-borne diseases, the modified mosquitoes present with undesirable traits, such as reduced life expectancy, delayed pupation, small-sized adults, and reduced performance compared to their unmodified counterparts (Bargielowski et al., 2011). The disadvantage of this method will be the cost employed for large scale engineering the mosquitoes (Vythilingam et al., 2016). Such approaches will increase the communication between the public and health practitioners as well as policymakers for potentially using genetically modified/engineered mosquitoes to combat infection caused by ZIKV (Adalja et al., 2016).

#### Sterile insect technique

Sterile insect technique (SIT) can also be employed to control the vector population. Several agriculturally important insect pests are sterilized using ionizing radiation from radioactive isotopes such as ^60^Co. However, these vectors are poor in health and are less effective in competing with wild type males. Therefore, the intended purpose of population control is not achieved. This technique is mainly used to control *Anopheles* sp. but it could also be employed for the control of *Aedes* sp. if basic information about their mating ecology is determined (Benelli, 2016). The strategy of using sterile males to induce sterility in wild fertile females can be used to reduce the insect population over a period of time. Using a suppression subtractive hybridization technique, 37 genes have been identified that are predominantly expressed in the testes of *Ae. aegypti*. A subset of 10 genes was chosen for RNAi-mediated knockdown to induce male sterility. It was observed that 9/10 knockdowns introduced male sterility in more than 50% of males, with reduced fecundity in the remaining insects (Whyard et al., 2015; Singh et al., 2016). In *Ae. aegypti* females, physiological and behavioral changes occur after mating. A subset of genes is activated to induce blood feeding and ovigenesis. Therefore, mating directly influences the vectoral capacity of *Ae. aegypti* by directing it to blood feeding and oviposition. Information regarding the post-mating changes in gene expression profile may be useful in developing strategies against the insect (Alfonso-Parra et al., 2016). Sterlization through γ-irradiation requires radio-isotopes and therefore poses health risk while handling it. Male sterilization through RNAi is though seemingly a promising strategy, major setback of this approach is the financial constraints faced during the large-scale application of this technique. Moreover, validation of the methodology adopted in the field is of utmost importance (Alphey et al., 2010; Weaver et al., 2016).

A pictorial representation of salient prevention and control measures of mosquito vector transmitting ZIKV is depicted in Figure 2.

#### Transmission blocking vaccines (TBVs)

Mosquitoes are developing resistance against the chemical insecticides that are commonly being used for their population control (Benelli, 2015). Hence, alternative strategies need to be designed and adopted for their efficient control. One of such recent approaches is the use of vaccines that can block the transmission of infectious agents from the vector (mosquito) to the host. These vaccines are termed as transmission blocking vaccines (TBVs) or “altruistic vaccines” (Londono-Renteria et al., 2016). The person who receives the vaccine may or may not be protected from the infectious agents but their neighbors will be protected; since the mosquitoes which bite the vaccinated person picks up the antibodies and thus the sites specific for infectious agent binding in the mosquitoes will be blocked by these antibodies (Londono-Renteria et al., 2016). Though, it seems weird that neighbors are only protected when the vaccine is administered to another person but due to the theory of herd immunity even the vaccinated person will also get protection in the long run (Dinglasan and Jacobs-Lorena, 2008). Although, this approach seems fascinating but it need assessment for its safety to the human health viz. autoimmune disorders (Kaslow, 1997). One important consideration to be take care is the maintenance of antibody titre in the vaccinated person as the antigen is not of human origin but is intended for mosquitoes (Coutinho-Abreu and Ramalho-Ortigao, 2010; Neelakanta and Sultana, 2015). To tackle this problem, suitable adjuvants can be employed such as IMX313 (a chicken complement C4b-binding protein oligomerization domain), exoprotein from *Pseudomonas aeruginosa* A and the outer membrane protein complex of *Neisseria meningitides* serogroup B (Qian et al., 2007; Li et al., 2016).

Presently, TBVs are under study for West Nile Virus and Dengue Virus, where these are designed to target the mosquito proteins that are essential for infection setup. Several targets have been identified for the development of TBVs against West Nile Virus like *Ae. aegypti* C-type lectin (mosGCTL-1) and similarly for Dengue Virus like mosGCTL-3, CRVP-379, CPB-1 (Isoe et al., 2009; Cheng et al., 2010; Colpitts et al., 2011; Bhatt et al., 2013; Liu et al., 2014; Tham et al., 2014). *In silico* analysis of three proteins, namely aegyptin, D7 and Sialokinin of *Ae. Aegypti*, was studied to identify B- and T-cell epitopes among these salivary proteins. These proteins are essential for the viral transmission between vector and host. Two B-cell epitopes LAALHVTAAPLWDAKDPEQF and TSEYPDRQNQIEE LNKLCKN were identified for D7 long form and D7 short form, respectively. Similarly, two T-cell epitopes MTSKNELDV and YILCKASAF for D7 long form and D7 short form, respectively, were identified (Sankar et al., 2017). These candidates can be further exploited for developing the TBVs that can prevent virus transmission to host. Eventhough, this technology is in its infancy, TBVs seems to be a promising avenue to prevent dreadful diseases that are transmitted through arthropods. Hence, such targets have to be timely identified for ZIKV so that TBVs can be developed for prevention of ZIKV transmission among the human population. Ultimately this approach will be especially beneficial for developing countries where the health care system is challenged economically (Londono-Renteria et al., 2016). The possible mechanism of TBVs has been picturized in Figure 1.

Different strategies to prevent and control mosquitoes are summarized in Table 1.

**Table 1:** Different strategies to prevent and control mosquitoes.

**Controlling modality**	**Controlling agent**	**Active against mosquito strain**	**Modus operandi**	**Remarks**	**Reference(s)**
Chemicals	N,N-diethyl-meta-toluamide (DEET) or picaridin containing pesticide	No specified strain tested	Curb *Ae. aegypti* mosquito population	N,N-diethyl-meta-toluamide (DEET) or picaridin- Safe to pregnant women Kline and Schutze, 2016
	Pyrethroids, organochloride, and organophosphorus		Curb general mosquito population by acting on nervous system of insect	Development of resistance and bioaccumulation are major problem	van den Berg et al., 2012
	Imidacloprid, thiacloprid, and thiamethoxam		Curb general mosquito population by larvicidal and adulticidal action		Uragayala et al., 2015
	IGRs (methoprene, pyriproxyfen, diflubenzuron, triflumuron)		Curb general mosquito population by larvicidal action (analogs juvenile hormone or inhibitors of chitin synthesis)		Mulla et al., 1986
	Picaridin		Binds to odorant binding protein 1 (AgamOBP1) and repel mosquitoes		Sluydts et al., 2016
Bacteria	*Wolbachia*	*Ae. Aegypti*	Decrease life span of female mosquito	Less chances of resistance development	Lambrechts et al., 2015; Nguyen et al., 2015
			Induce cytoplasmic incompatibility		Aliota et al., 2016
	*Bacillus thuringiensis israelensis*	*Aedes, Anopheles, Coquillettidia, Culex*, and *Mansonia* sp.	Bacterial toxins kill various species of mosquito		Kollars, 2016; Singh et al., 2016
Fungus	*Metarhizium anisopliae*	*Ae. aegypti*	Fungal hyphae invasion into internal organs of mosquito and death of the host (adulticidal effect)	No chance of resistance development Cost effective method	Darbro and Thomas, 2009
	*Beauveria bassiana*		Fungal toxin released cause death		Tiago et al., 2014
	*Aspergillus nomius*	*Ae. Albopictus*	Fungal penetration in insect body		Jaber et al., 2016
Mosquito	*Toxorhynchites splendens*	*Ae. aegypti*	Mosquito larvae predation		Brown et al., 1996; Benelli et al., 2016a
Copepods	*Mesocyclops thermocyclopoide*	*Ae. aegypti*	Feed on 1st instar larvae	Copepod rearing is cheap Little assistance is required for maintenance of the colonies	Schaper and Hernández-Chavarría, 2006; Mahesh Kumar et al., 2012
	*Macrocyclops albidus*	*Ae. albopictus* and *Cx. pipiens*			
	*Megacyclops formosanus*	*Anopheles* sp. and *Cx. quinquefasciatus*	Feed on 1st and 2nd instar larvae		Kalimuthu et al., 2014
	*M. aspericornis*	*Ae. aegypti*	Feed on 1st instar larvae		Murugan et al., 2013
	*Mesocyclops aspericornis*	*Cx.quinquefasciatus*	Feed on 1st instar larvae		Nathiya et al., 2015
Herbs	*Myracrodruon urundeuva* Fr. Allemao	*Ae. aegypti*	Seed extract toxic to mosquito	No chances of resistance development or bioaccumulation Often less toxic to the other living species sharing the same micro environment	Souza et al., 2011
	*Aegle marmelos* (Linn.), *Limonia acidissima* (Linn.), *Sphaeranthus indicus* (Linn.)	*Cx. quinquefasciatus* and *Ae. aegypti*	Ovicidal		Reegan et al., 2015
	*Apium graveolens, Callistemon rigidus, Persea americana* extract	*An. gambiae, Ae. aegypti* and *Cx. quinquefasciatus*	Larvicidal		Kumar et al., 2014; Pierre et al., 2014; Ramkumar and Karthi, 2015
	*Clusia fluminensis*	*Ae. aegypti*.			Anholeti et al., 2015
	*Syzygium lanceolatum*	*An. stephensi, An. subpictus, Ae. aegypti, Ae. albopictus, Cx. quinquefasciatus*, and *Cx. tritaeniorhynchus*.			Benelli et al., 2016b
	*Cleistanthus collinus* and *Murraya koeingii* hexane extracts	*Cx. quinquefasciatus*			Tennyson et al., 2012
	*Ichnocarpus frutescens*	*An. subpictus, An. albopictus*, and *Cx. Tritaeniorhynchus*			Govindarajan et al., 2016b
	*Heracleum sprengelianum*				Govindarajan and Benelli, 2016
	Leaf extract of *E. coronaria*	*Cx. quinquefasciatus, Ae. aegypti* and *An. stephensi*	Ovicidal and repellent		Mathivanana et al., 2010
	*Origanum scabrum*	*An. stephensi, Ae. aegypti, Cx. quinquefasciatus*, and *Cx. tritaeniorhynchus*	Larvicidal and ovicidal		Govindarajan et al., 2016a
	*Moringa oleifera*	First to fourth instar larvae of the *An. stephensi*	Larvicide and Pupicide		Prabhu et al., 2011
	*Glycosmis pentaphylla*	*An*. *stephensi, Cx*. *quinquefasciatus* and *Ae*. *aegypti*	Larvicide and adulticide		Ramkumar et al., 2016
Fishes	*Gambusia affinis*	*Anopheles* sp. *Aedes* sp.	Larvicidal	Mosquito larvae are main target thus halting the development of mosquitoes at initial stage Imbalance in native flora and fauna after introducing non-native predatory fishes	Kant et al., 2013; Sarwar, 2015
	*C. gariepinus*	fourth instar larvae of *An. stephensi*	Larvicidal and adulticidl		Ghosh et al., 2005
	*Cyprinus carpio, Ctenopharyngodon idella, Oreochromis niloticus* and *Clarias gariepinus*	*An. stephensi, An. subpictus* Grassi, *Armegeres subalbatus* Coquillett, *Cx. quinquefasciatus* and *Cx. vishnui*			
	*Poecilia reticulate*	*Cx. quinquefasciatus, Ae. aegypti, Ae. albopictus*			
	*Panchax minnow*				
	*Psuedomugil signifer, Hyseleotris galii, Pseudogobius* sp.	*Ae. vigilax, Cx. annulirostris*	Larvicidal		Griffin, 2014
	*Channa gachua*	Not specified	Larvicidal		Phukon and Biswas, 2013
	*Oreochromis niloticus* (Tilapias)				Chandramohan et al., 2016
	*Carassius auratus* (goldfish)				
	Beta Splendens (Siamese fighting fish)				
	Dormitator latifrons (Pacific fat sleeper),				
Tadpoles	*Bufo, Euphlyctis, Hoplobatrachus, Polypedates* and *Ramanella*	*Ae. aegypti* egg	Ovicidal	Sharing same environment and thus effectively used as mosquito control strategy	Bowatte et al., 2013
	*Hoplobatrachus tigerinus*	*Ae. aegypti*	Larvicidal		Murugan et al., 2015b
Genetic tailoring	*Ae. aegypti* OX513A	*Ae. aegypti*	Competition with wild type mosquitoes for food and reproduction	Though promising, but are costly and laboriou.	Phuc et al., 2007; Bargielowski et al., 2011; Alphey and Alphey, 2014
Sterile insect technique	*Anopheles* sp. (Co irradiation)	*Anopheles* sp.	Irradiated male competes with wild type males		Benelli, 2016
	A subset of 10 genes having testicular origin knockdown by RNAi-mediated to make then sterile	*Ae. aegypti*	Sterile male or males with reduced fecundity compete with wild type males		Whyard et al., 2015; Singh et al., 2016

### Prevention and control strategies for human ZIKV infection

Apart from controlling the vector population that is involved in the transmission of ZIKV, it is also essential to adopt preventive measures at the individual level by the use of vaccines or employing strategies interfering the non-vectoral transmission of ZIKV. Nonetheless, as of now there is no commercial ZIKV vaccine in the market, but owing to the rapid pace of research in vaccine development, time is not far away for the availability of an effective ZIKV vaccine.

#### Vaccines against ZIKV infection

The major strategy that can be employed for the effective control of ZIKV infection is the use of vaccine, which is yet in its primitive stage but is gaining high interest among researchers worldwide. Several vaccine platforms are currently under way for the development of an effective vaccine to prevent ZIKV infection (Asif et al., 2017; Barzon and Palù, 2017; Fernandez and Diamond, 2017; Munjal et al., 2017a). Due to the alarming nature of ZIKV and its potential threat to human population, within nearly 2 year of announcing ZIKV as an emergency by WHO, more than 40 vaccine candidates have been reported to be under preclinical study, 7 are in phase I trial and one is in its phase 2b trial. Of note, the hemagglutinin subunit 1 (HA1/ H1) protein of influenza virus pdmH1N1 and the E protein of ZIKV shares similarity in frequency component (0.295) and thus the neutralization of ZIKV can happen by the antibodies generated against the pdmH1N1 H1 protein, ultimately affecting the cellular entry of ZIKV. Thus, proposal had been put forward to employ influenza vaccine (seasonal) consisting of the component of pdmH1N1 in order to prevent the spread of ZIKV (Veljkovic and Paessler, 2016; Zhao et al., 2016). The recent advancements in designing and developing vaccines against ZIKV are presented in the following section.

##### Inactivated vaccines

Inactivated vaccines are prepared through the killing of the pathogenic organism and immunizing host along with an adjuvant. Inactivated vaccines can be given to even immune compromised individuals; however, these require repeated immunizations to achieve the protective antibody titers. Inactivated vaccines are available for several flaviviruses such as Yellow Fever virus, West Nile fever virus, Japanese encephalitis virus, and others. An India based vaccine manufacturer, Bharat Biotechnologies, Hyderabad, India has initiated the efforts to develop inactivated ZIKV vaccine in early 2013 before the ZIKV disease emerged to Brazil as an epidemic and it is currently in its phase I clinical trial (Sumathy et al., 2017). There was protection of monkeys against Asian ZIKV strain which had prior exposure to African lineage prototype MR766 ZIKV strain. This observation explored the possibility of development of the vaccines that can protect against all lineages of ZIKV (Dowd et al., 2016a; Dyer, 2016). This African lineage prototype MR766 is in the pipeline for development of an inactivated ZIKV vaccine. Another formalin-inactivated vaccine named ZIKV purified inactivated virus (ZPIV), developed by Walter Reed Army Institute, United States of America (USA), displaying better protective efficacy in monkeys and mice, is also in phase I clinical trial (Larocca et al., 2016). An experimental study of this vaccine with AG 129 mice showed that there was complete protection against challenge with both homotypic and also heterotypic ZIKV strains. Phase I clinical studies conducted on 67 participants employing ZPIV revealed seroconversion after administration of 2 doses of the vaccine and there was minimal side effect (Modjarrad et al., 2017). Further studies are warranted for employing the new generation vaccine adjuvants and also to improve the immunogenicity before the inactivated ZIKV vaccine can hit the commercial market.

##### Live attenuated ZIKV vaccine

Live attenuated vaccines are made by weakening the natural virus or bacteria using heat, chemical or genetic manipulation. Such vaccines modulate both the arms of the immune system, humoral and cell mediated, and even with fewer doses sufficient level of protection is obtained. A Cambodian ZIKV strain candidate (FSS13025) with deletion of 10 nucleotides in the 3′ untranslated region was developed to create a live attenuated ZIKV vaccine. A study in A129 mice (type I interferon-deficient mice) showed that the developed vaccine candidate was completely attenuated, elicited immunity and rendered protection against ZIKV challenge (Shan et al., 2017a). Another study with the live attenuated ZIKV vaccine trial in the pregnant mice indicated that there was minimal level of ZIKV RNA in placenta, maternal and fetal tissues on 6 and 13 days of embryonic life after vaccination. Similarly, vaccination of male mice also protected testicular damage and oligospermia due to ZIKV (Shan et al., 2017b). Recently, a patent (WO2017156511A1) has been granted for the development of live attenuated ZIKV vaccine (Whitehead et al., 2017). Codagenix, New York, USA, reported the development of ZIKV vaccine (CDX-ZKV) employing codon deoptimization technology. A chimeric ZIKV vaccine employing pre-membrane (prM) and Envelope (E) genes of ZIKV in a backbone of attenuated DENV2 is under construction to fight against ZIKV infection (Morabito and Graham, 2017).

##### DNA based vaccines

Nucleic acid vaccine designing is the promising platform that is in limelight during the recent years for the progressive development of an effective ZIKV vaccine. DNA vaccines coding prM-E protein genes of ZIKV have been developed and are in clinical trial. Inovio Pharmaceuticals, Pennsylvania, USA, has developed a DNA vaccine, GLS-5700, which is the first ZIKV vaccine to enter the clinical trial. Vaccine Research Center (VRC), National Institute of Allergy and Infectious Diseases, National Institutes of Health, USA has also developed two ZIKV DNA vaccines, namely VRC5288 (ZIKV and Japanese Encephalitis chimeric vaccine) and VRC5283 (ZIKV prM-E vaccine), which are in phase I clinical studies (Morabito and Graham, 2017). Phase I study conducted with 80 participants for VRC5288 and 45 participants for VRC5283 revealed that these vaccines are safe with minimal side effects. The participants (14 nos.) who received split dose vaccine of VRC5283 had 100% antibody response and based on such encouraging results VRC5283 was suggested for next level clinical trial (Gaudinski et al., 2017). As per the recent report, VRC5283 has now entered phase IIb clinical trial. Both these DNA vaccines provided better immunity and controlled viremia in 17 out of 18 rhesus monkeys when tested along with another vaccine candidate VRC8400 (Dowd et al., 2016b). Though, DNA vaccine strategy appears fascinating, few limitations are also associated with it such as the need for large quantity of DNA to immunize and further there may be a risk of its integration into the host genome.

##### mRNA based vaccines

The mRNA vaccine is another type of nucleic acid based vaccine which gets translated immediately after its entry into the cell, unlike DNA vaccine that needs entry to the nucleus for initial transcription followed by the translation. The mRNA vaccines have emerged as better alternative to the DNA vaccine, as these does not integrate with the host genome and thus offers higher safety in comparison to the DNA vaccine. Such a vaccine may be directly transfected to cytoplasm so can evade problems associated with the nuclear delivery. The mRNA based vaccines have several other advantages like easy delivery with liposomes, cationic polymers (Fotin-Mleczek et al., 2011), gene gun and electroporation (Cu et al., 2013), and these binds to pattern recognition receptor and can act as self-adjuvant (Fotin-Mleczek et al., 2011). Nucleoside-modified mRNA encompassing ZIKV glycoprotein (preM-E) enveloped in lipid nanoparticles has been shown to induce strong humoral response and initiate T-cell immunity in mice and macaques with a single immunization using 30 μg of ZIKV prM–E mRNA–LNP construct (Richner et al., 2017). The use of nanoparticles here ensured the intracellular delivery of mRNA, a critical requirement for a potential vaccine candidate. The mRNA based self-replicating technology also may be developed, but its limitation that vaccine itself may produce innate immune response and toxicity must be addressed before use. A single epitope IGVSNRDFV from the ZIKV E protein has been recently identified, which is conserved across all the ZIKV clades and to which strong CD8+ T-cell response was found to be induced in immunized C57BL/6 mice (Chahal et al., 2017). This approach provides an excellent mechanism to develop a vaccine without requiring a recombinant glycoprotein or reference virus stock and may also have the potential to be generated readily at the time of outbreaks in emergency situations. More recently, a long-lasting immunity has been reported to be provided in non-human primates by employing mRNA-LNP (lipid nanoparticle-encapsulated nucleoside modified mRNA) thereby appearing to be a putative candidate vaccine in the coming years (Pardi et al., 2017; Richner et al., 2017).

##### Vectored vaccine

Vectored vaccine is another approach for the development of ZIKV vaccines. Recently, measles vector based ZIKV vaccine has been developed by Themis Biosciences expressing the ZIKV prM-E proteins. Under experimental condition, a single dose of this vectored vaccine protected monkey from two different strains of ZIKV and the vaccine is presently in its preclinical studies. Due to the alarming concern about the antibody-dependent enhancement (ADE) of Dengue/ZIKV as a result of the use of ZIKV envelope immunogens as vaccine candidates, there is a search for an alternative vaccine strategy. Thus, viral vectored vaccine expressing ZIKV NS1 can be a promising alternative to prevent the ADE (Brault et al., 2017). Modified vaccinia Ankara vectored ZIKV expressing NS1 and Vesicular Stomatitis Virus vectored ZIKV expressing prM-E have been developed by Geovax and Harvard, respectively. An effective long-term durable protection provided by such vaccine is essential in order to fight against ZIKV infection. As, most of the challenge studies were carried out following ZIKV vaccines during the peak immune status; hence, a clear picture regarding the durability of the vaccines is not known. Immunization of rhesus monkeys with an adeno vectored ZIKV vaccine followed by dual immunization with purified inactivated ZIKV vaccine provided protective immune response even at 1 year of vaccination (Abbink et al., 2017). Thus, this approach can provide immunity against ZIKV atleast for 1 year, which can be further studied to know the exact protection period. An adenovirus serotype 5-vectored vaccine- Ad5.ZIKV-Efl (virus vectored) has also been recently developed that is under clinical consideration (Kennedy, 2016; Kim et al., 2016). Other approaches like subunit vaccine are also being evaluated for the development of ZIKV vaccine by different firms like PaxVax, VBI Vaccines, and NewLink Genetics (Morabito and Graham, 2017).

##### Cytotoxic T lymphocyte vaccine

Cytotoxic T lymphocyte (CTL) response constitutes an imperative arm of the host defense system to render protection against any of the infectious pathogens. CD4+ T-cell epitope-based DNA vaccine has been found highly immunogenic and to elicit the significant protective response. Human Leukocyte Antigen—antigen D Related (HLA-DR) is a major histocompatibility class II (MHC II) cell surface receptor, which along with 9-amino acid long stretch of peptide/epitope forms a ligand for T-cell receptor (TCR). Such peptide sequences may be predicted using the different algorithms like TEPITOPE and ProPred. By this approach “promiscuous” CD4+ T-cell epitopes from the conserved E and M proteins of ZIKV can be predicted which are able to bind with multiple HLA-DR molecules, and hence could pave way for developing a CTL based vaccine against this virus (Cunha-Neto et al., 2017).

##### Lysosome-associated membrane protein vaccine

Carboxyl terminal domain of the protein associated with the lysosome-associated membrane protein (LAMP) is used to direct the antigen to the MHC II vesicular compartment of the antigen-presenting cells. LAMP1 and LAMP2 are the most abundant lysosomal proteins. These are essential for maintaining membrane integrity of the lysosome and release of hydrolytic enzymes (Jiang et al., 2015). The cytoplasmic tail of LAMP-1 is comprised of 11 amino acids (RKRSHAGYQTI), and in particular the last 4 amino acid residues (YQTI), are determinant for the proteins' lysosomal targeting (Lu, 2003). A DNA chimera constructs having West Nile virus preM-E and LAMP when used to immunize mice, it elicited significant antibody and long-term neutralization titers in comparison to those mice that were immunized with the construct expressing the untargeted antigen (Anwar et al., 2005). Immunization with a DNA construct having Yellow fever virus (YFV) matrix and envelop protein fused with LAMP sequence showed higher neutralization titers, that protected mice from intracerebral challenge with the YFV virus (Dhalia et al., 2009). Although, no attempt has been made to develop such LAMP based vaccine for ZIKV, in near future such vaccine may be developed with proper efficacy verification of the vaccine.

##### Computer-aided vaccine designing

It is another platform that led to the identification of various ZIKV candidate targets like E, NS3 and NS5 proteins which can be employed for development of peptide vaccines (Usman Mirza et al., 2016). An *in silico* analysis carried out on the ZIKV NS1 gene revealed that it is conserved and distributed among all different isolates of ZIKV. Of note, this study resulted in the identification of NS1 of ZIKV that has the potential to elicit the immune response thereby preventing ZIKV infection (Dos Santos Franco et al., 2017). Thus, it makes a hope for commercially viable effective Zika vaccine in the near future to prevent ZIKV infection among the human population. Nevertheless, molecular dynamic studies are needed to analyse the immunogenic CTL epitopes for the presentation of MHC-antigen as well as to evaluate its stability. Docking of approximately 15 CTL epitopes (conformational) with multiple MHCI proteins has been done recently. Side-by-side simulation of the conditions was also performed for the purpose of their interaction (Usman Mirza et al., 2016). The results would be enormously helpful for designing the peptide based vaccines as the data may provide with the preliminary bunch of peptides. Among the ZIKV T-cell epitopes certain peptides viz., QTLTPVGRL and IRCIGVSNRDFV are found highly antigenic in nature (Ashfaq and Ahmed, 2016).

**Figure 3:**
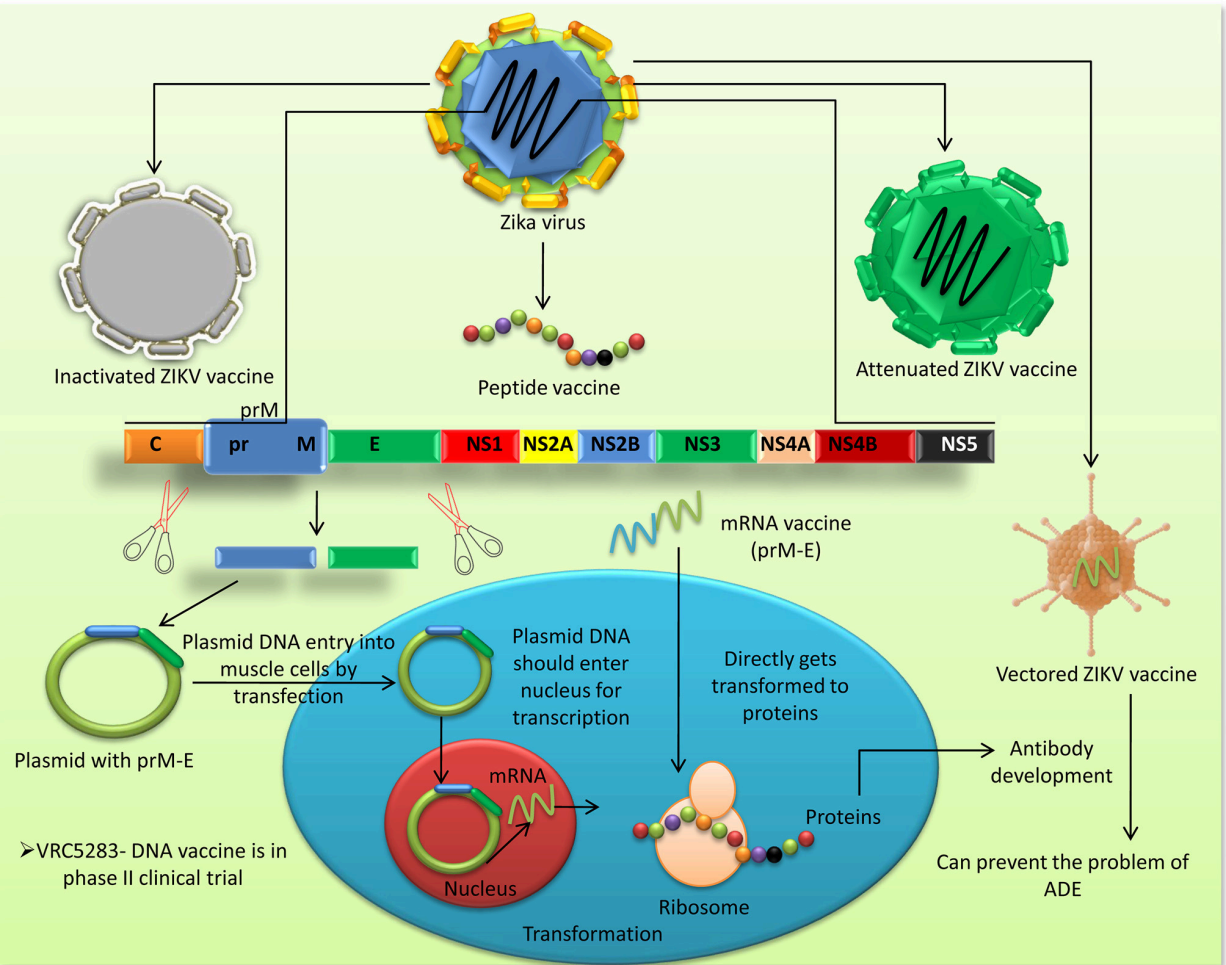
Different vaccine platforms available for prevention and control of ZIKV infection in humans.

Figure 3 summarizes the different vaccine platforms available for ZIKV prevention and control.

#### Other prevention and control strategies

Transmission through the vectors is not the sole way of the ZIKV spread. There are several other routes (non-vector borne) which must be taken into consideration in order to design the control strategies against the spread of this virus. As far as the issues related to complications of health is concerned there must be general awareness among the public so that basic cleanliness measures can be adapted to confirm elimination of breeding space for vector larvae (Grischott et al., 2016). ZIKV is reported to transmit sexually, hence safe sexual contact is a pre-requisite for prevention of viral infection and also sexual intercourse is not advised for 6 months from the time of onset of signs in male partners or from the time of diagnosis (Musso et al., 2015; Mead et al., 2017). Practice of safe sex in high risk areas should be considered as an important precautionary measure. It is advisable to avoid sexual intercourse if traveling in such an area or if one partner is infected with ZIKV (Rather et al., 2017). Those planning to conceive after their visit to endemic areas must wait at least 28 days to allow a 2 week incubation period and another 2 weeks for the period of viremia to end. Visitors from such areas must be kept under surveillance for symptoms for at least 1 month (Maharajan et al., 2016; Singh et al., 2016; Krow-Lucal et al., 2017). Parental care and use of contraceptives should be provided in ZIKV-endemic countries to minimize the incidence of ZIKV-associated microcephaly (Sharma and Lal, 2017). As ZIKV is known to be present in semen (Mansuy et al., 2016) and saliva (D'Ortenzio et al., 2016), therefore, the use of contraceptives is emphasized (Atkinson et al., 2016; D'Ortenzio et al., 2016; Mansuy et al., 2016). Tests conducted for the persistence of ZIKV in biological fluids revealed that only semen remains positive for ZIKV-RNA after 27 and 62 days (Atkinson et al., 2016). However, a subsequent study showed the prolonged presence of ZIKV in semen as long as 144 days after the onset of clinical symptoms (Huits et al., 2017). Similarly, another study showed the persistence of ZIKV in semen even on 188 days after the onset of symptoms in an Italian traveler returning from Haitii (Nicastri et al., 2016).

Although, the presence of infective ZIKV particles has been reported in breast milk (Dupont-Rouzeyrole et al., 2016), there is no report of its transmission to neonates. As it is presumed that the advantages of breast feeding are greater than the possible risk of ZIKV transmission and therefore, the CDC has recommended that infected mothers and mothers living in endemic areas continue to breast feed their neonates. Blood transfusion can also disseminate ZIKV if preventive measures have not been taken timely (Vasquez et al., 2016). Thus, inactivation of the virus by the way of pasteurization and solvent/detergent treatment along with virus removal using filters with a 40 nm pore size, can effectively reduce viral load in the plasma-derived medicinal products (Blümel et al., 2016; Farcet and Kreil, 2017; Kühnel et al., 2017). Amotosalen combined with UV light has also shown to inactivate ZIKV in fresh or frozen plasma (Aubry et al., 2016; Santa Maria et al., 2017). ZIKV has been reported to be inactivated for infectious particles by 6.57 log_10_ TICD_50_/mL and for viral RNA by 10.25 log_10_ copies/mL in frozen plasma by using the combination of amotosalen and UV light (Aubry et al., 2016; Musso et al., 2017). Recently, amustaline (S-303) and glutathione (GSH) have been identified to inactivate ZIKV in red blood cells (Laughhunn et al., 2017). The high risk group of ZIKV includes health workers, hence hygienic practices should be employed to minimize the risk of spread of ZIKV within the inmates of the hospital (Rather et al., 2017).

In the initial week following ZIKV infection, bites of *Aedes* mosquitoes should be avoided. At the same time, it is advisable for the patients to remain under bed-nets. The health workers must take precautionary measures to avoid infection of hospitalized patients from such health workers. Use of insect repellant like *N, N*-diethyl-3-methylbenzamide, 3-(*N*-butyl-*N*-acetyl) amino propionic acid ethyl-ester (icaridin) should be encouraged (Sikka et al., 2016; Rather et al., 2017). People traveling to endemic areas must be properly educated about the use of mosquito repellents and mosquito nets during their travel. Pregnant women must avoid visiting such areas due to the risk of brain malformation of the fetus. If they have already traveled to an endemic region, they must be kept under proper medical supervision. Surveillance and monitoring should be at its highest level at airports, harbors, and other ports of entry to prevent entry of ZIKV from endemic countries (Marano et al., 2016). Mosquitoes control programs combined with surveillance study within 200 meters of ZIKV infected persons at South Korea revealed that there were no cases of ZIKV after vector control measures. Hence, monitoring and surveillance is of utmost importance for the prevention and control of Zika disease (Chang et al., 2017).

Last but not the least, especially the societies which are vulnerable to the emerging threats of ZIKV infection should be addressed appropriately by the policy makers at the government level. The current global trend of climate changes that influences the vector density cannot be ignored at this time. In developing countries, certain crucial measures viz., advance planning and development of infrastructure prior to the disease outbreaks are often neglected that make the situation grave. So, such priorities should be set timely in this regard for efficient prevention and control ZIKV infection and limiting its massive spread. Nevertheless, a sense of responsibility and collective efforts at the level of both government as well as the public are the need of hour (Dasti, 2016; Mourya et al., 2016).

**Figure 4:**
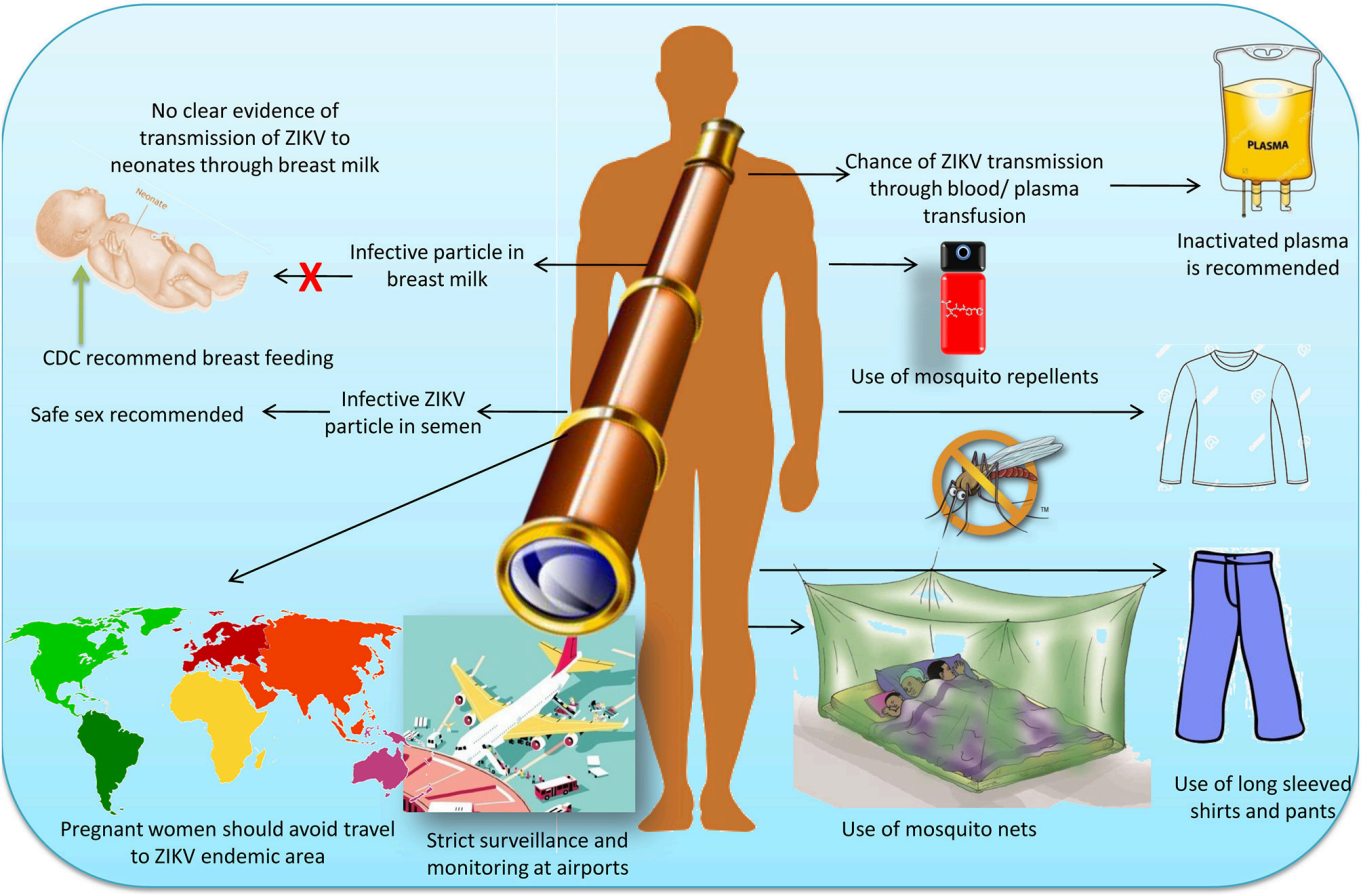
Possible ways of Zika virus transmission, its prevention and control measures in humans.

The possible ways of ZIKV transmission, its prevention and control measures in humans are indicated in Figure 4.

## Conclusions and future perspectives

The involvement of the *Aedes* mosquito in the transmission of ZIKV has gained global attention. High density human population, lack of immunity and mutations in the virus helps in the propagation of the virus. Additionally, global warming has acted as a catalyst. Eventhough, there are many modes of ZIKV transmission, priority must be given to the control of mosquitoes. Hence, more focus is needed to adopt appropriate mechanical, biological and chemical measures to prevent or inhibit their breeding and thus, control the mosquito population. Use of chemicals has the disadvantages of development of resistance and bioaccumulation; hence in the recent time, a shift has been seen toward biopesticides. Several plant extracts and essential oils are found effective against mosquito control. The spread of viruses by mosquitoes can be checked using bacteria such as *Wolbachia pipientis* and alpha-proteobacterium, which may transfer both horizontally and vertically. Genetic engineering techniques using siRNA to knock down testes-specific genes, resulting in male mosquito sterility, may also be employed. The use of fungi to control the *Ae. aegypti* mosquito seems to be promising, but further research is needed to determine the viability, infectivity, and spore persistence in the field. The use of fish seems to be a good option but due care should be taken in selecting the kind of fish for predation, as non-native species can cause problem of invasive behavior, and spoiling aquatic microenvironment.

Though, currently there is no commercial vaccine available in the market against ZIKV, however presently much advancement in this direction have been initiated with promising outcomes by several group of researchers worldwide for the development of an effective vaccine. Several vaccine candidates are now in preclinical and clinical phase studies and expected to reach the market soon with possibilities to prevent ZIKV infection in humans. Recently, the use of transmission blocking vaccines (TBV) preventing the spread of disease from infected and vaccinated person to naïve person have shown great promise. If employed successfully then the whole community can be benefited as the non-vaccinated person also gets protection. Exploiting the fields of immune profiling, bioinformatics, cloud computing, vaccinomics, reverse immunology and other advances to their full potential applications would ultimately make possible the designing and developing of efficient, more convenient and cost effective ZIKV vaccines in the near future.

The use of genetically modified vectors has many benefits, but it will be important to educate government authorities, public health officials, and scientists regarding the effectiveness of this strategy. Further research is required to analyze the cases of ZIKV infection to determine the relationship between the virus and genetic mutations of the *Aedes* mosquito. This will increase our understanding of the mechanisms of the transmission cycle of the virus and the reasons for favoring this specific mosquito. Genetically modified mosquitoes, which are not potent to reproduce and can compete with wild type mosquitoes are needed. However, the location must be taken into consideration before conducting such small-scale experiments in the field. The use of mosquito-repelling herbal products should also be encouraged, since these are safe for any age and sex and could be used by pregnant women without fear of adverse effects. Health workers must be protected to avoid the spread of infection to hospitalized patients and utmost care must be taken while donating blood or organs.

It must be noted that globalization has facilitated the rapid spread of ZIKV infection in large scale and to widespread territories worldwide. The infectious agent is allowed to thrive well due to change in climate globally along with overcrowding in the urban areas. So, it is high time to re-think about these issues and rebuild our infrastructure as far as the public health is concerned and to develop effective strategies to control the disease. Apart from the well-established mosquito-borne mode of ZIKV transmission, there is need to converge our focus on the other modes of transmission of ZIKV, as yet our knowledge regarding the other possible modes is hazy. This would help to carve out better prevention and control strategies against ZIKV. There is need to advise pregnant mothers to avoid traveling to ZIKV infection endemic areas, educate the public regarding the disease outcome and progress so far, and to add to the current epidemiological data. Lastly, but importantly, researchers and scientists must share data on the status of mosquito population control between countries. The ZIKV cases should be studied in detail in order to check the correlation between the infectious agents and mutation in the *Aedes* mosquito population. This, in turn, will help to understand the reasons for this particular type of mosquitoes being the carrier of ZIKV infection. Side by side the development of the vaccine should be carried out at a rapid pace so as to reach the commercial market soon.

## Author contributions

All the authors substantially contributed to the conception, design, analysis and interpretation of data, checking and approving final version of manuscript, and agree to be accountable for its contents. RS, KD, RK, and AM initiated this review compilation; RK designed tables and KK designed the figures; SC, AM, KK, and RT updated different intervention strategies; YM and RS reviewed virological aspects and analyzed data; KD and RB-M reviewed, analyzed, and edited.

### Conflict of interest statement

The authors declare that the research was conducted in the absence of any commercial or financial relationships that could be construed as a potential conflict of interest.
